# Environmental enteropathy and malnutrition: do we know enough to intervene?

**DOI:** 10.1186/s12916-014-0187-1

**Published:** 2014-10-14

**Authors:** William A Petri, Caitlin Naylor, Rashidul Haque

**Affiliations:** Division of Infectious Diseases & International Health, University of Virginia, Charlottesville, VA 22908-1340 USA; icddr,b, GPO Box 128, Mohakhali, Dhaka 1000 Bangladesh

**Keywords:** Environmental enteropathy, Mesalazine, Inflammation, Malnutrition

## Abstract

Environmental enteropathy (EE) is a poorly defined state of intestinal inflammation without overt diarrhea that occurs in individuals exposed over time to poor sanitation and hygiene. It is implicated as a cause of stunting and malnutrition, oral vaccine failure and impaired development in children from low-income countries. The burden on child health of malnutrition alone, which affects 25% of all children and is estimated to result in more than a million deaths annually due to heightened susceptibility to infection, makes urgent a solution to EE. Efforts are thus underway to treat EE even while work continues to identify it through the use of non-invasive biomarkers, and delineate its pathogenesis. A recent study published in *BMC Medicine* reports the first randomized controlled phase I trial of an anti-inflammatory drug for EE. The aminosalicylate mesalazine was found to be safe in short-term treatment of a small number of severely malnourished children, although efficacy was not established. Whether such treatment trials are premature, or instead a way both to understand and intervene in EE, is the focus of this article.

Please see related article: http://www.biomedcentral.com/1741-7015/12/133.

## Background

Environmental enteropathy (EE) is an inflammatory condition of the gut of residents of low-income countries that is a result of exposure to poor sanitation and hygiene [1-4]. The cause of EE is postulated to be inflammation from continuous fecal-oral exposure to enteropathogens. It is defined pathologically by decreased villous height and lymphocytic infiltration in the small intestinal lamina propria and epithelium. It has been measured functionally by abnormalities in sugar absorption using the lactulose:mannitol test. Biomarkers of EE include intestinal inflammation, gut barrier dysfunction and intestinal epithelial health. Consequences of EE are hypothesized to include linear growth faltering, impaired child development and oral vaccine failure [1,2]. There is even the thought that the current knowledge gap in the prevention and treatment of malnutrition, where current interventions are modeled to be less than one third effective, may be due to EE (Table 1) [5].

**Table 1:** **What works for the prevention and treatment of malnutrition**

**Intervention**	**Application**
Improving women’s nutrition, especially before, during and after pregnancy	Mother
Early and exclusive breastfeeding for first 6 months	Mother-infant
Timely, safe, appropriate good quality complementary feeding for 6-24 months	Infant
Micronutrient supplementation or fortification (iron, zinc, vitamin A, iodine, folate, calcium, vitamin D)	Mother & infant
Promotion of responsive infant feeding practices	Mother
Treatment of severe acute malnutrition	Infant

**Figure 1:**
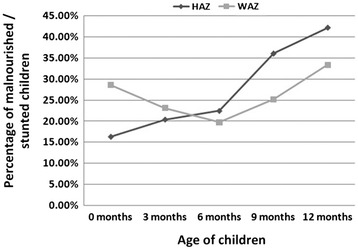
**Percentage of Bangladesh birth cohort infants malnourished (WAZ ≤2) and stunted (HAZ ≤2) from birth to 12 months.** Height-for-age Z (HAZ) and weight-for-age Z (WAZ) scores determined using the World Health Organization’s Anthro software, version 3.0.1 [3].

Linear growth faltering occurs within the first two years of life and then, for the most part, is irreversible, necessitating early diagnosis if prevention or treatment is to be successful (Figure 1) [6,7]. There is, therefore, emphasis on identification of biomarkers that will detect EE when it is still a subclinical illness. There are several candidate biomarkers for which there is limited evidence of usefulness. These include markers of gut inflammation such as fecal neopterin and myeloperoxidase, of gut barrier dysfunction including fecal alpha-1- antitrypsin, of intestinal mannitol absorption, and intestinal epithelial regeneration measure Reg1 (Table 2) [1,2,8,9]. It is a fair summary of the state of current knowledge that EE is characterized by intestinal injury and both gut and systemic inflammation.

**Table 2:** **Biomarkers for studies of environmental enteropathy**

**Biomarker**	**Indicative of**
Reg1 (fecal)	Intestinal epithelial health
IGF-1 (plasma)	Growth and proliferation
Calprotectin (fecal)	Enteric inflammation
Myeloperoxidase (fecal)	Enteric inflammation
Neopterin (fecal)	Enteric inflammation
Alpha-1 anti-trypsin (fecal)	Intestinal barrier disruption
Lipopolysaccharide (plasma)	Intestinal barrier disruption
Lactulose:mannitol (urine)	Intestinal barrier disruption
sCD14 (plasma)	Systemic inflammation
CRP	Systemic inflammation
ferritin	Systemic inflammation
IL-6	Systemic inflammation
IL-1b	Systemic inflammation

CRP, c-reactive protein; IGF, insulin-like growth factor; IL, interleukin.

### Mesalazine as a treatment for environmental enteropathy

A study recently published in *BMC Medicine* by Jones *et al*. [4] represents a first step in testing the role of anti-inflammatory treatment for EE, in this case in the setting of severe acute malnutrition. Therapy based on suppression of inflammation is based on the hypothesis that the inflammation of EE is deleterious, much as it is in high-income countries for inflammatory bowel diseases, such as Crohn’s, ulcerative colitis and celiac disease. This, of course, is not necessarily the case, as the inflammation may be protective against the enormous burden of enteric infection suffered by these children [10].

The authors demonstrate the safety of oral mesalazine in one- to five-year-old children undergoing treatment for severe acute malnutrition in a slum in Nairobi, Kenya. The study was conducted in the summer and fall of 2013. Forty-four children of average age 19 months, who were undergoing treatment for severe acute malnutrition (SAM) and who had evidence of EE, were randomized to receive mesalazine or placebo. EE for the purposes of the study was defined as a height-for-age z-score ≤2 and systemic inflammation (as measured by an erythrocyte sedimentation rate >20 mm/hour). Intestinal inflammation was not a criterion for inclusion, although it was present in 95% of the children, as evidenced by a fecal calprotectin level higher than 100 μg/g. Enrollment criteria included being between one and five years of age and having uncomplicated SAM (defined by mid-upper arm circumference <11.5 cm or bilateral pedal edema). Exclusion criteria included severe clinical illness, lack of appetite and need for inpatient nutritional therapy. The most common illnesses that resulted in exclusion were hepatic dysfunction (elevation of liver enzymes was seen in 8% of those screened), malaria and a need for inpatient treatment.

All children received nutritional rehabilitation with ready-to-use therapeutic food, deworming with mebendazole or albendazole, and a seven-day course of amoxicillin. Therapy continued until cure, as measured by mid upper arm circumference >11.5 cm and no edema at two consecutive weekly visits. Mesalazine was administered at 30 mg/kg/day for days 1 to 7, and in the absence of identified toxicity, was escalated to 45 mg/kg/day for a further 21 days. The study was a double-blinded and randomized placebo-controlled trial. Follow-up was for a total of 56 days. Primary outcomes were adverse events and compliance with the intervention.

At completion of the 56-day follow-up period, nineteen children (34%) had not recovered, had died or had not remained in follow-up. There were no significant differences between the mesalazine and placebo groups in any measure of toxicity or efficacy. There was a lower erythrocyte sedimentation rate (ESR) and a trend towards lower fecal calprotectin (*P* = 0.09) and immunoglobulin G (IgG) Endocab antibodies (*P* = 0.07) at the end of 28 days of mesalazine treatment, but this was not sustained at 56 days. There was no difference in other inflammatory markers such as CRP, endotoxin, sCD14 platelets or white cell count. There was also no difference in nutritional outcome, although the rate of increase in mid-upper arm circumference was higher in the placebo arm of the study. In all children studied, systemic endotoxin was negatively, and IGF-1 positively, associated with linear growth, supporting a role of gut barrier dysfunction in inflammation-induced stunting.

**Figure 2:**
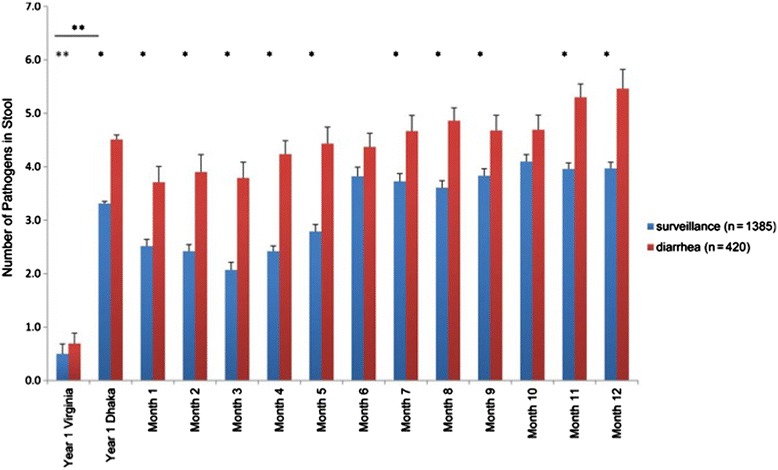
**Frequency of enteropathogen detection in infants in Dhaka versus Virginia.** Diarrheal and nondiarrheal stool samples were collected at the time points indicated and assayed for 29 enteropathogens by molecular methods. The total number of enteropathogens was summed for each sample; results are shown as mean ± SE. *Bonferroni adjusted *P* value <0.05 (determined with a linear mixed-effect regression model used to identify differences in the number of pathogens detected between diarrheal and surveillance samples for each month during the study period). **Nonparametric Wilcoxon 2-sample tests were used to compare numbers of pathogens between Virginia and Dhaka samples and between diarrheal and surveillance samples for Virginia alone [10]. SE, standard error.

Perhaps the most important finding was that mesalazine was safe in this small study of malnutrition treatment in infants. The safety of aminosalicylates, such as mesalazine, is attractive in the setting of treatment of already ill children who are suffering from SAM and multiple enteric infections [10]. The doses used were comparable to those used to induce remission for mild to moderate Crohn’s disease. This is a potentially important finding, as one could have anticipated a worsening of symptoms if inflammation was, in fact, protective via its action against enteropathogens in the gut (Figure 2). However, this conclusion may be premature, as treatment had no demonstrable effect on gut, and only a transient effect on systemic, inflammation.

The lack of any demonstration of efficacy of mesalazine could be for several reasons. First, a more prolonged treatment may be required, or at an earlier age (the average age of the children treated was 19 months, a time at which the majority of stunting has already occurred). However even in high-income countries these agents are effective at inducing remission in less than half of patients with ulcerative colitis and are not recommended for inducing remission of Crohn’s disease [11].

### Other treatment options for environmental enteropathy

There are other therapeutic options for EE that are being considered. These include antibiotics, probiotics, other anti-inflammatory agents, drugs targeted to tight junction regulation, and epithelial healing. A study of the broad-spectrum and relatively unabsorbed antibiotic rifaximin was conducted in asymptomatic three- to five-year-old children in rural Malawi [12]. The average HAZ of the children was – 1.7 and 76% had an abnormally elevated lactulose:mannitol ratio at the time of enrollment. Children were randomized in a double-blinded, placebo-controlled trial of 100 mg twice daily of rifaximin or placebo for seven days. Twenty-eight days after the initiation of treatment, the children were restudied with the lactulose:mannitol test. No significant difference was observed in the sugar absorption test. Treatment with rifaximin was complicated by a significant increase in diarrhea (13%), a toxicity which may preclude future trials with this agent.

Budesonide is a synthetic corticosteroid that is produced in an enteric-coated formulation that results in delivery of the drug to the ileum and ascending colon where it has an anti-inflammatory effect. In clinical trials budesonide has induced remission in up to a fifth of patients with ulcerative colitis [13]. Long-term use has unfortunately been complicated by decreased bone mineral density, suggesting significant systemic uptake of the drug leading to toxicity. α4β7 antagonists to prevent migration of inflammatory lymphocytes to the gut are another potential, but currently untested, therapeutic approach [14].

Regulation of tight junction permeability by zonulin, which is upregulated in celiac disease, is a potential therapeutic target. Larazotide acetate is an octapeptide that is a competitive inhibitor of the receptor binding domain of zonulin and in early clinical trials has been shown to decrease intestinal permeability following gluten challenge in celiac disease patients [14].

A different approach from anti-inflammatories is to aid repair of the damaged gut with glutamine derivatives. Glutamine is estimated to comprise a third of respiratory metabolism in the gut and may be most readily administered in dipeptide derivatives such as alanyl-glutamine [15]. Glutamine supplementation was tested for its impact on intestinal barrier function in Brazil in a study of moderately and severely malnourished hospitalized children (WAZ ≤2). The children were between 2 and 60 months of age. Fifty-three children were randomly assigned to receive in double-blind fashion standard formula supplemented either with glutamine or as a control glycine. The formula was administered for 10 days. The outcome measure was a change in the lactulose/mannitol test for intestinal barrier function. Supplementation with glutamine significantly improved (that is, lowered) the lactulose:mannitol ratio between day 1 and day 10 [15].

Likely at the heart of the problem of intestinal inflammation and malnutrition is the gut microbiota. Current probiotics are not of proven efficacy for any intestinal condition and have not been tested for EE [16]. On the horizon though is the potential for next generation probiotics. A recent study examined the composition of the microbiota in malnourished Bangladeshi children. A microbiota maturity index was defined by measuring the composition of the microbiota, based on 16S ribosomal RNA sequencing, in a cohort of children living in an urban slum of Dhaka who had consistently healthy growth. Children with severe acute malnutrition had significantly immature microbiota, which was partially restored by nutritional therapy. A total of 24 taxa of bacteria were identified that were most age-discriminatory [17]. One can imagine in the near future that EE might be prevented or treated by restoration of key bacteria present in the gut of healthy children.

Epigenetic contributions must also be considered. While most stunting occurs after birth, there is a substantial contribution of maternal nutritional status to the ultimate outcome of the child, suggesting a role for epigenetics. In fact, recently it has been shown that maternal methyl donor pathway biomarkers are predictive of infant nutritional status and are associated with DNA methylation of metastable epialleles [18]. Interventions aimed at improving the nutritional status of the mother are already of known effectiveness, and could be acting in part via epigenetics.

Finally, intestinal damage due to mycotoxins from fungal contamination of food may also contribute to stunting [19]. Aflatoxin, for example, causes intestinal damage including increased leakiness of the gut barrier, and aflatoxin-albumin adducts in plasma were associated with stunting in a dose-dependent manner in one study. If validated at other sites, then this would suggest an intervention aimed at protecting food production from mycotoxin contamination.

The study by Jones *et al*. comes at a time when major clinical studies of EE are nearing completion. These include the Mal-ED study of malnutrition and enteric diseases and the PROVIDE study of the impact of EE on oral vaccine failure, both supported by the Bill & Melinda Gates Foundation. These studies are defining the measures of the enteropathy that results from gut infection in infants, and estimating its impact on nutrition, vaccination and child development. These studies have the promise of informing studies of treatment or prevention of EE.

## Conclusions

Anti-inflammatory agents are one proposed therapeutic approach to EE. While it is increasingly clear that EE is characterized, in part, by gut and systemic inflammation, it is not clear if that inflammation is deleterious in the way that it is for inflammatory bowel disease, or, conversely, protective against the near universal infections of children with enteric pathogens. The study by Jones and colleagues is encouraging for the lack of toxicity observed with mesalazine, although tempered by the fact that the anti-inflammatory effect of the drug was modest. There was a small decrement in the ESR but no effect on the gut as measured by fecal calprotectin. It, therefore, remains an open question of whether anti-inflammatory therapy will be safe or effective but the future is bright. A new understanding of EE is forthcoming with the completion of multi-site observational studies of infants in low-income countries, and a better understanding of pathogenesis is accompanying biomarker discovery. As therapeutic trials begin for EE, the use of new biomarkers for gut homeostasis, injury, inflammation and repair promise to illuminate outcomes. Likely only through rationally designed therapeutic trials with emphasis on safety first, and with the use of biomarkers to judge response, will we arrive at a treatment for this most important of diseases.

## Abbreviations

EE: environmental enteropathy
HAZ: height for age Z score
SAM: severe acute malnutrition
WAZ: weight for age Z score
